# Lymphoma and metastatic breast cancer presenting with palpable axillary and inguinal lymphadenopathy in a 40-year-old man with rheumatoid arthritis on anti-tumor necrosis factor α therapy: a case report

**DOI:** 10.1186/1752-1947-7-23

**Published:** 2013-01-15

**Authors:** Gourab Datta, Dorendra Maisnam

**Affiliations:** 1 Breast Surgery, St. Albans Hospital, West Hertfordshire Hospitals NHS Trust, Saint Albans, Hertfordshire, UK; 2 Department of Histopathology, Hemel Hempstead Hospital, Hemel Hempstead, Hertfordshire, UK

**Keywords:** Breast cancer, Humira®, Lymphoma, Rheumatoid arthritis, TNFα

## Abstract

**Introduction:**

We present the case of a 40-year-old man with severe rheumatoid arthritis being treated with high-dose anti-tumor necrosis factor α therapy (adalimumab), who developed simultaneous lymphoma and breast cancer with lymph node metastases. We describe strategies for investigations and management of this presentation.

**Case presentation:**

A 40-year-old Caucasian man with severe rheumatoid arthritis being treated with high-dose adalimumab presented to our facility with a swollen leg and palpable left groin and left axillary lumps and a left nipple lesion. Left lower limb ultrasound, computed tomography and positron emission tomography scans showed extensive lymphadenopathy. Core biopsies of the left groin, axilla and nipple lesion showed this to be concurrent diffuse B-cell lymphoma and locally metastatic invasive ductal carcinoma of the breast. He underwent a left mastectomy with axillary clearance, and adjuvant fluorouracil, epirubicin and cyclophosphamide chemotherapy with rituximab, and the adalimumab was stopped.

**Conclusions:**

The findings from our patient’s case should increase awareness that patients with severe rheumatoid arthritis, especially if they are on high-dose biological treatments, have the potential to develop lymphoma, which in turn increases the risk of developing other primary tumors, so that in rare cases a patient may have concurrent tumors. Assessment and management of these patients is challenging and should include computed tomography scans of the of neck, thorax, abdomen and pelvis, including a fludeoxyglucose positron emission tomography/computed tomography scan, bone marrow testing and appropriate core biopsies and discussion at multidisciplinary team meetings about treatment of the separate tumors in the presence of hematologists, oncologists, surgeons and rheumatologists.

## Introduction

We present the case of a Caucasian man with rheumatoid arthritis (RA) who was being treated with adalimumab (Humira®, Abbott Laboratories, Global Health Care and Medical Research, 100 Abbott Park Road, Abbott Park, Illinois, USA), an anti-tumor necrosis factor α (TNFα) biological agent, who developed synchronous B-cell lymphoma and locally metastatic breast cancer. RA is a chronic inflammatory condition resulting in destructive arthropathy of joints. The pathophysiology involves an autoimmune activation of T helper lymphocytes, antigen-presenting cells and production of cytokines such as TNFα acting against joint synovium. The advent of biological therapies, which inhibit inflammatory pathways, have had success in the treatment of RA.

The immunosuppressive effects of biological agents such as adalimumab have the potential concern of increasing the risk of lymphoma, although the evidence for this in RA is controversial, as severe cases of RA are usually on adalimumab and increased disease severity itself is an increased risk factor of developing lymphoma.

Lymphoma is a hematological cancer affecting the lymphatic system. The lymphocytes are cancerous and functionally abnormal resulting in an immunosuppressed state. This is associated with an increased risk of developing other types of primary malignancies ranging from colorectal cancer to breast cancer [1].

This case report highlights that when patients with RA who have been on biological agents present with unusual symptoms it is necessary to consider lymphoma and a synchronous second primary neoplasm, and suggests diagnostic and therapeutic strategies for addressing this situation.

## Case presentation

A 40-year-old Caucasian man with a background of severe seropositive RA affecting his shoulders, wrists and left hip was worked up for an elective total hip replacement (THR). He had a medical history of RA, diagnosed 14 years previously, for which he had been taking adalimumab (two weekly injections) for seven years, methotrexate 20mg weekly, salazopyrin 1g twice a day and prednisolone 1mg once a day. He had no other medical conditions and no family history of cancer. He was a former heavy smoker (quit four years ago, 40 pack-years) and drank 24 units of alcohol per week. On admission for his left THR, pre-operatively it was discovered that his entire left leg had been swollen for three weeks. He reported no other symptoms, including systemic symptoms of weight loss or night sweats. The operation was cancelled. An ultrasound scan of the left lower limb showed no deep vein thrombosis, but multiple abnormal looking lymph nodes in the groin were seen. He went on to have a computed tomography (CT) scan of his thorax, abdomen and pelvis. This demonstrated extensive lymphadenopathy affecting axillae, inguinal regions, mediastinum and retroperitoneum. The lymph nodes were particularly bulky in the left axilla, retroperitoneum and left inguinal regions. The CT scan also showed a left nipple mass.

Our patient was referred to a hematologist and a breast surgeon for specialist assessment. The history was as noted above. On examination, added to the swollen left leg, bilateral axillary lymph nodes were palpable which were more bulky on the left, a 5 × 3cm hard left groin lymph node was palpable and a nodular grape-like pink lesion 2cm in diameter was seen over the left nipple. There was no hepatosplenomegaly palpable. Our patient’s adalimumab injections were stopped permanently after consultation with a rheumatologist. Our patient underwent core biopsies of the left nipple lesion, left axilla, left groin and bone marrow biopsy. Histology and immunostaining studies showed the left nipple lesion was an intermediate grade invasive ductal carcinoma of the breast with no lympho-vascular invasion. The groin biopsy showed a relatively homogeneous lymphoid cell population with mainly small lymphocytes demonstrating atypia suspicious for lymphoma. Test results revealed the cells were CD20+, CD5 negative, B-cell lymphoma (bcl)-6 positive and bcl-2 positive, in keeping with a B-cell lymphoma. The bone marrow biopsy was essentially normocellular except for a single paratrabecular cluster of lymphoid cells containing CD20+ B cells and CD3+ T cells suspicious for a follicular lymphoma. Fluorescence *in situ* hybridization (FISH) carried out on the groin lymph node core biopsy sample was inconclusive, with inconsistent translocations of bcl-2 and many cells showing an amplification of part of chromosome 18, the overall significance of which was uncertain, but could not confirm or exclude that this was a follicular B-cell lymphoma. The core biopsy of the left axilla sampled three separate lymph nodes which contained metastatic adenocarcinoma of the breast.

**Figure 1:**
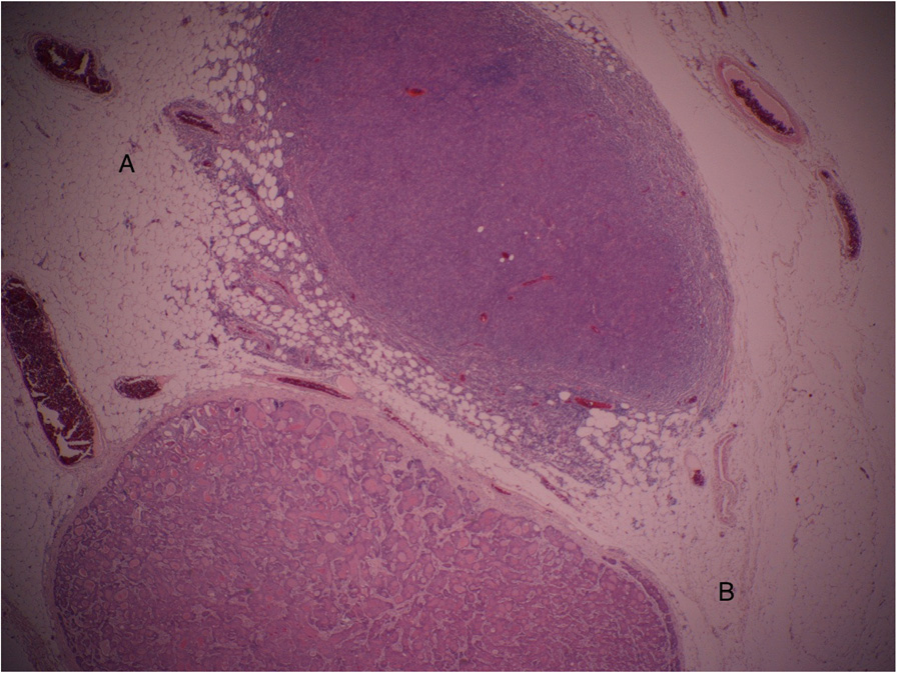
**(A) axillary lymph node replaced by lymphoma. (B)** Second lymph node from same axillary lymph node specimen showing second lymph node replaced by metastatic invasive ductal carcinoma of the breast.

Our patient went on to have a fludeoxyglucose (FDG) positron emission tomography (PET)-CT scan from the base of the skull to the mid-thighs. This showed a focal intense uptake in the left breast in the region of the known breast cancer, and increased uptake patterns consistent with extensive nodal disease above and below the hemidaiphragm, particularly bulky in the retroperitoneum and left inguinal region, consistent with the original CT scan. Increased uptake in the axilla and supraclavicular regions was seen, but it was not possible from the PET-CT scan to assess whether they represented nodes from lymphoma or metastatic breast cancer. All these results were discussed at the hemoncology and breast multidisciplinary team meeting and a decision was made to proceed with a left mastectomy and axillary clearance. The histology of this showed a 22mm low-grade invasive ductal carcinoma with prominent lymphatic invasion and numerous microcalcifications with clear excision margins and no evidence of Paget’s disease of the nipple. The tumor tested positive for estrogen receptor (8/8) and positive for progesterone receptor (6/8), but negative for human epidermal growth factor receptor 2 (Her-2). The left axillary contents contained 20 lymph nodes, five out of 20 nodes contained metastatic breast adenocarcinoma, all the others contained lymphoma. There was one lymph node, half of which was involved with metastatic breast cancer, and the other half with lymphoma (Figure 1). Our patient went on to have adjuvant chemotherapy to cover the breast cancer and lymphoma consisting of a regimen of fluorouracil, epirubicin and cyclophosphamide (FEC) chemotherapy with rituximab and thromboprophylaxis with enoxaparin 40mg subcutaneously once a day. 

## Discussion

In the current report we describe the case of a 40-year-old Caucasian man with severe RA diagnosed 14 years previously who had been treated with high-dose adalimumab for seven years, who presented with a swollen leg and palpable left groin and left axillary lumps and a left nipple lesion. Left lower limb ultrasound, CT and PET scans showed extensive lymphadenopathy. Core biopsies of the left groin, axilla and nipple lesion showed this to be concurrent diffuse B-cell lymphoma and locally metastatic invasive ductal carcinoma of the breast. He underwent a left mastectomy with axillary clearance, adjuvant FEC chemotherapy with rituximab and the adalimumab was stopped. When abnormal lymph nodes are demonstrated clinically with or without ultrasound scanning in a patient with RA on biological treatment such as adalimumab, it is reasonable to do full staging with a CT scan of the neck, thorax, abdomen and pelvis, with directed core biopsies to assess the extent and nature of the lymphadenopathy and to guide management.

In our patient’s case it is difficult to ascertain whether severe long-term RA alone or adalimumab treatment of RA was the risk factor that contributed to development of lymphoma. It is recognised that RA increases the risk of developing lymphoma, especially for severe and longstanding disease, although the precise mechanism for this is not understood. Adalimumab is an anti-TNFα antibody, which is a biological therapy for RA. It is difficult to assess the contribution of anti-TNFα therapy to developing lymphoma, as patients with severe RA are the same patients on anti-TNFα therapy. Evidence from observational cohort studies using more than 15,000 patients found no increased risk of lymphoma or any solid tumors in patients with RA receiving anti-TNFα treatment, although RA was more severe in the group receiving the biological treatment. In another cohort study with over 1500 patients with RA after adjusting for age, sex and disease severity there was a small increased risk (hazard ratio 5.0) of developing lymphoma in the RA group treated with a TNFα inhibitor (five lymphomas in treatment RA group versus two lymphomas in the reference RA group) [2]. This finding has to be interpreted with caution because of the small number of lymphomas (a total of seven) and short follow-up period of four years [3]. In response to reports of lymphomas through post-marketing surveillance, the Food and Drug Administration (FDA) summarised data from clinical trials for all TNFα inhibitors. They found that lymphomas were rare occurrences in both treatment and placebo groups with a trend towards a small increase in the treatment group, which was not statistically significant. Bongartz *et al*. conducted a meta-analysis of nine randomized controlled trials (RCTs) of anti-TNFα treatment of RA [4]. The odds ratio (OR) for overall malignancy was 2.4 (95% confidence interval [CI] 1.2 to 4.8) with skin cancer being the most common malignancy and lymphoma the second most common with 10 cases in the treatment group and zero cases in the placebo group. The risk of malignancy was dose related with high-dose TNFα inhibitor therapy having an increased OR of 4.3 (95% CI 1.6 to 11.8). In summary, neither has a causal relationship between anti-TNFα therapy for RA and lymphoma been established or ruled out. Severe longstanding RA does increase the risk of developing lymphoma. In our patient’s case it is impossible to say what the causative factor was, but it is likely to have been either RA or adalimumab [5].

In our patient’s case he had also been taking methotrexate. This is an immunosuppressant agent. The question of whether such agents increase the risk of malignancy in RA has been previously addressed. A cohort study of 789 randomly selected patients with RA between 1999 and 2005 found that methotrexate did not increase the risk of cancer although other immunosuppressant agents did increase the risk [6]. A cohort study of patients with RA found that there was increased risk of malignant melanoma with methotrexate with a standardized incidence ratio of 3.0 (95% CI 1.2 to 6.2) [7], although another group found no increased risk of skin cancer with methotrexate in a study of 15,789 patients with RA [8]. A review of this subject found that the use of methotrexate in patients with RA did not increase the risk of cancers, although azathioprine increased the risk of developing lymphomas and cyclophosphamide increased the risk of cancers, particularly bladder cancer [5]. The current evidence indicates that there is no increased risk of developing non-cutaneous malignancies with the use of methotrexate in RA.

Lymphoma increases the risk of developing other primary cancers, as the abnormal lymphocytes result in a functionally immunosuppressed state. Immunosuppression increases the risk of developing other primary malignancies, as a functioning immune system can detect abnormal epitopes on the surface of some cancer cells, which activates an immune response to destroy those cells. In our patient’s case it is likely that lymphoma contributed to the development of breast cancer, as this was a man with no family history of cancer. Once lymphoma has been identified on initial imaging and histologically, it is reasonable to do a FDG PET-CT scan not only to assess the extent of disease but also to identify possible locations of other primary tumors. Once diagnosis of lymphoma is confirmed, a PET-CT scan to assess the extent of lymphoma and identify any other occult primary tumor is already considered good practice [1]. This proved to be so in our patient’s case, where the primary breast cancer was identified.

This case report should increase awareness that patients with severe RA, especially if they are on high-dose biological treatments, have the potential for developing lymphoma, which in turn increases the risk of developing other primary tumors so that in rare cases a patient may have concurrent tumors. Assessment and management of these patients is challenging and should include a CT scan of the neck, thorax, abdomen and pelvis, including an FDG PET-CT scan, bone marrow test and appropriate core biopsies and discussion at multidisciplinary team (MDT) meetings about treatment of the separate tumors in the presence of hematologists, oncologists, surgeons and rheumatologists. Our patient’s case was discussed at five MDT meetings during our patient’s treatment process.

## Conclusions

Patients with severe RA on anti-TNFα treatment may in rare cases develop concurrent lymphoma and another primary tumor. This should be considered in all such patients undergoing investigation and management for either a lymphoma or another primary tumor so as not to miss the concurrent tumor and an opportunity to treat both. These decisions should be made in the setting of MDT meetings incorporating all the involved specialists.

## Consent

Written informed consent was obtained from the patient for publication of this case report and any accompanying images. A copy of the written consent is available for review by the Editor-in-Chief of this journal.

## Competing interests

The authors declare that they have no competing interests.

## Authors’ contributions

GD analyzed and interpreted the data from patient records and was a major contributor in writing the manuscript. DM performed the histological examination of all specimens from our patient and selected appropriate figures. Both authors read and approved the final manuscript.

## References

[B1] Papajík T Mysliveček M Sedová Z Buriánková E Procházka V Raida L Kubová Z Neoral C Starostka D Mikula P Melichar B Kučerová L Tichý M Indrák K Synchronous second primary neoplasms detected by initial staging F-18 FDG PET/CT examination in patients with non-Hodgkin lymphoma Clin Nucl Med 2011 36 509 512 10.1097/RLU.0b013e318217541d 21637049

[B2] Geborek P Bladstrom A Turesson C Gulfe A Petersson IF Saxne T Olsson H Jacobsson LT Tumour necrosis factor blockers do not increase overall tumour risk in patients with rheumatoid arthritis, but may be associated with an increased risk of lymphomas Ann Rheum Dis 2005 64 699 703 10.1136/ard.2004.030528 15695534 PMC1755491

[B3] Franklin JP Symmons DP Silman AJ Risk of lymphoma in patients with RA treated with anti-TNFalpha agents Ann Rheum Dis 2005 64 657 658 10.1136/ard.2005.035311 15834052 PMC1755469

[B4] Bongartz T Sutton AJ Sweeting MJ Buchan I Matteson EL Montori V Anti-TNF antibody therapy in rheumatoid arthritis and the risk of serious infections and malignancies: systematic review and meta-analysis of rare harmful effects in randomized controlled trials JAMA 2006 295 2275 2285 10.1001/jama.295.19.2275 16705109

[B5] Beauparlant P Papp K Haraoui B The incidence of cancer associated with the treatment of rheumatoid arthritis Semin Arthritis Rheum 1999 29 148 158 10.1016/S0049-0172(99)80026-2 10622679

[B6] Abasolo L Judez E Descalzo MA Gonzalez-Alvaro I Jover JA Carmona L Group ES Cancer in rheumatoid arthritis: occurrence, mortality, and associated factors in a South European population Semin Arthritis Rheum 2008 37 388 397 10.1016/j.semarthrit.2007.08.006 17977580

[B7] Buchbinder R Barber M Heuzenroeder L Wluka AE Giles G Hall S Harkness A Lewis D Littlejohn G Miller MH Ryan PF Jolley D Incidence of melanoma and other malignancies among rheumatoid arthritis patients treated with methotrexate Arthritis Rheum 2008 59 794 799 10.1002/art.23716 18512713

[B8] Chakravarty EF Michaud K Wolfe F Skin cancer, rheumatoid arthritis, and tumor necrosis factor inhibitors J Rheumatol 2005 32 2130 2135 16265690

